# 4-Chloro-*N*-(2,5-dimethyl­phen­yl)-2-methyl­benzene­sulfonamide

**DOI:** 10.1107/S1600536811040876

**Published:** 2011-10-08

**Authors:** Vinola Z. Rodrigues, Sabine Foro, B. Thimme Gowda

**Affiliations:** a Department of Chemistry, Mangalore University, Mangalagangotri 574 199, Mangalore, India; b Institute of Materials Science, Darmstadt University of Technology, Petersenstrasse 23, D-64287 Darmstadt, Germany

## Abstract

The asymmetric unit of the title compound, C_15_H_16_ClNO_2_S, contains three independent moleules. The conformation of the N—H bonds are *anti* to the *ortho*-methyl groups of the sulfonyl benzene rings in all the mol­ecules. The sulfonyl and the aniline benzene rings are tilted relative to each other by 43.0 (2), 37.0 (2) and by 46.0 (1)° in the three mol­ecules. In the crystal, inter­molecular N—H⋯O hydrogen bonds link each of the mol­ecules into centrosymmetric dimers.

## Related literature

For the preparation of the title compound, see: Savitha & Gowda (2006 ▶). For hydrogen-bonding modes of sulfonamides, see: Adsmond & Grant (2001 ▶). For studies on the effects of substituents on the structures and other aspects of *N*-(ar­yl)-amides, see: Gowda *et al.* (2000 ▶), on *N*-(ar­yl)-methane­sulfonamides, see: Gowda *et al.* (2007 ▶), on *N*-(ar­yl)-aryl­sulfonamides, see: Gelbrich *et al.* (2007 ▶); Perlovich *et al.* (2006 ▶); Rodrigues *et al.* (2011 ▶); Shetty & Gowda (2005 ▶) and on *N*-(chloro)-aryl­sulfonamides, see: Gowda & Shetty (2004 ▶).
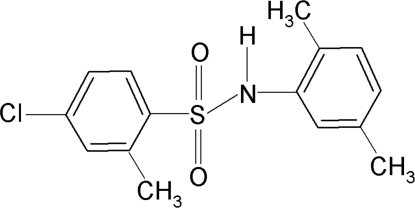


## Experimental

### 

#### Crystal data

C_15_H_16_ClNO_2_S
*M* *_r_* = 309.80
Triclinic, 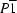

*a* = 10.092 (1) Å
*b* = 12.585 (1) Å
*c* = 18.523 (2) Å
α = 96.404 (9)°
β = 95.279 (9)°
γ = 103.39 (1)°
*V* = 2257.4 (4) Å^3^
*Z* = 6
Mo *K*α radiation
μ = 0.39 mm^−1^
*T* = 293 K
0.46 × 0.28 × 0.20 mm

#### Data collection

Oxford Diffraction Xcalibur diffractometer with a Sapphire CCD detector
Absorption correction: multi-scan (*CrysAlis RED*; Oxford Diffraction, 2009 ▶) *T*_min_ = 0.840, *T*_max_ = 0.926
15326 measured reflections
8214 independent reflections
3972 reflections with *I* > 2σ(*I*)
*R*_int_ = 0.033

#### Refinement

*R*[*F* ^2^ > 2σ(*F* ^2^)] = 0.070
*wR*(*F* ^2^) = 0.152
*S* = 1.02
8214 reflections
559 parameters
3 restraints
H atoms treated by a mixture of independent and constrained refinement
Δρ_max_ = 0.30 e Å^−3^
Δρ_min_ = −0.27 e Å^−3^

### 

Data collection: *CrysAlis CCD* (Oxford Diffraction, 2009 ▶); cell refinement: *CrysAlis RED* (Oxford Diffraction, 2009 ▶); data reduction: *CrysAlis RED*; program(s) used to solve structure: *SHELXS97* (Sheldrick, 2008 ▶); program(s) used to refine structure: *SHELXL97* (Sheldrick, 2008 ▶); molecular graphics: *PLATON* (Spek, 2009 ▶); software used to prepare material for publication: *SHELXL97*.

## Supplementary Material

Crystal structure: contains datablock(s) I, global. DOI: 10.1107/S1600536811040876/bt5662sup1.cif

Structure factors: contains datablock(s) I. DOI: 10.1107/S1600536811040876/bt5662Isup2.hkl

Supplementary material file. DOI: 10.1107/S1600536811040876/bt5662Isup3.cml

Additional supplementary materials:  crystallographic information; 3D view; checkCIF report

## Figures and Tables

**Table 1:** Hydrogen-bond geometry (Å, °)

*D*—H⋯*A*	*D*—H	H⋯*A*	*D*⋯*A*	*D*—H⋯*A*
N1—H1*N*⋯O2^i^	0.83 (2)	2.15 (2)	2.958 (4)	164 (4)
N2—H2*N*⋯O6^ii^	0.85 (2)	2.15 (2)	2.951 (5)	157 (4)
N3—H3*N*⋯O3^iii^	0.85 (2)	2.13 (2)	2.962 (5)	166 (4)

Symmetry codes: (i) 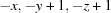; (ii) 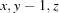; (iii) 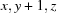.

**Fig. 1.**
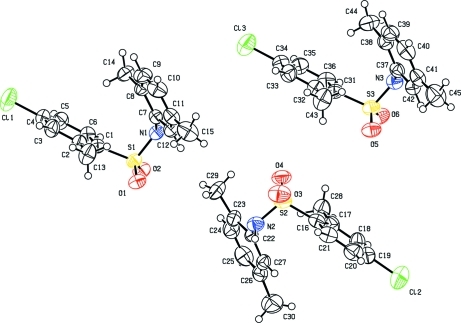
Molecular structure of the title compound, showing the atom labelling scheme and displacement ellipsoids are drawn at the 50% probability level.

**Fig. 2.**
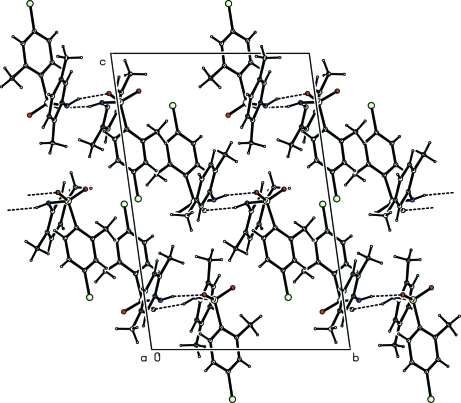
Molecular packing of (I) with hydrogen bonding shown as dashed lines.

## supplementary crystallographic information

### Comment

The amide and sulfonamide moieties are the constituents of many biologically significant compounds. The hydrogen bonding preferences of sulfonamides have been investigated (Adsmond & Grant, 2001). As part of our work on the substituent effects on the structures and other aspects of *N*-(aryl)-amides (Gowda *et al.*, 2000), *N*-(aryl)-methanesulfonamides (Gowda *et al.*, 2007), *N*-(aryl)-arylsulfonamides (Rodrigues *et al.*, 2011; Shetty & Gowda, 2005) and *N*-(chloro)-arylsulfonamides (Gowda & Shetty, 2004), in the present work, the crystal structure of 4-Chloro-2-methyl-*N*-(2,5-dimethylphenyl)benzenesulfonamide (I) has been determined (Fig. 1).

The asymmetric unit of (I) contains three independent moleules. The conformation of the N—H bonds are *anti* to the *ortho*-methyl groups in the sulfonyl benzene rings of all the molecules.

The torsion angles of the C—SO_2_—NH—C segments in the three molecules of (I) are 66.8 (3)°, -68.7 (4)° and 76.9 (4)°, compared to the values of -66.8 (3)° and 70.3 (3)° in the two independent molecules of 4-chloro-2-methyl-*N*-(2,3-dimethylphenyl)benzenesulfonamide (II) (Rodrigues *et al.*, 2011).

The sulfonyl and the aniline benzene rings in (I) are tilted relative to each other by 43.0 (2)° in molecule 1, 37.0 (2)° in molecule 2 and 46.0 (1)° in molecule 3, compared to the values of 44.1 (1)° and 39.7 (1)° in the two independent molecules of (II).

The other bond parameters in (I) are similar to those observed in (II) and other aryl sulfonamides (Perlovich *et al.*, 2006; Gelbrich *et al.*, 2007).

In the crystal, the intermolecular N–H···O hydrogen bonds (Table 1) link the molecules to centrosymmetric dimers. Part of the crystal structure is shown in Fig. 2.

### Experimental

The solution of *m*-chlorotoluene (10 ml) in chloroform (40 ml) was treated dropwise with chlorosulfonic acid (25 ml) at 0 ° C. After the initial evolution of hydrogen chloride subsided, the reaction mixture was brought to room temperature and poured into crushed ice in a beaker. The chloroform layer was separated, washed with cold water and allowed to evaporate slowly. The residual 2-methyl-4-chlorobenzenesulfonylchloride was treated with 2,5-dimethylaniline in the stoichiometric ratio and boiled for ten minutes. The reaction mixture was then cooled to room temperature and added to ice cold water (100 cc). The resultant solid 4-chloro-2-methyl-*N*- (2,5-dimethylphenyl)-benzenesulfonamide was filtered under suction and washed thoroughly with cold water. It was then recrystallized to constant melting point from dilute ethanol. The purity of the compound was checked and characterized by recording its infrared and NMR spectra (Savitha & Gowda, 2006).

Prism like colourless single crystals used in X-ray diffraction studies were grown in ethanolic solution by slow evaporation at room temperature.

### Refinement

The H atoms of the NH groups were located in a difference map and their coordinates were refined with the  N—H distance restrained to 0.86 (2) %A. The other H atoms were positioned with idealized geometry using a riding model with the aromatic C—H = 0.93Å and methyl C—H = 0.96 Å. All H atoms were refined with isotropic displacement parameters. The *U*_iso_(H) values were set at 1.2*U*_eq_(C-aromatic, N) and 1.5*U*_eq_(C-methyl).

### Crystal data

**Table:**

C_15_H_16_ClNO_2_S	*Z* = 6
*M**_r_* = 309.80	*F*(000) = 972
Triclinic, *P*1	*D*_x_ = 1.367 Mg m^−^^3^
Hall symbol: -P 1	Mo *K*α radiation, λ = 0.71073 Å
*a* = 10.092 (1) Å	Cell parameters from 2462 reflections
*b* = 12.585 (1) Å	θ = 2.6–27.9°
*c* = 18.523 (2) Å	µ = 0.39 mm^−^^1^
α = 96.404 (9)°	*T* = 293 K
β = 95.279 (9)°	Prism, colourless
γ = 103.39 (1)°	0.46 × 0.28 × 0.20 mm
*V* = 2257.4 (4) Å^3^	

### Data collection

**Table:**

Oxford Diffraction Xcalibur diffractometer with a Sapphire CCD detector	8214 independent reflections
Radiation source: fine-focus sealed tube	3972 reflections with *I* > 2σ(*I*)
graphite	*R*_int_ = 0.033
Rotation method data acquisition using ω scans	θ_max_ = 25.4°, θ_min_ = 2.6°
Absorption correction: multi-scan (*CrysAlis RED*; Oxford Diffraction, 2009)	*h* = −12→11
*T*_min_ = 0.840, *T*_max_ = 0.926	*k* = −15→15
15326 measured reflections	*l* = −21→22

### Refinement

**Table:**

Refinement on *F*^2^	Primary atom site location: structure-invariant direct methods
Least-squares matrix: full	Secondary atom site location: difference Fourier map
*R*[*F*^2^ > 2σ(*F*^2^)] = 0.070	Hydrogen site location: inferred from neighbouring sites
*wR*(*F*^2^) = 0.152	H atoms treated by a mixture of independent and constrained refinement
*S* = 1.02	*w* = 1/[σ^2^(*F*_o_^2^) + (0.0494*P*)^2^ + 1.3294*P*] where *P* = (*F*_o_^2^ + 2*F*_c_^2^)/3
8214 reflections	(Δ/σ)_max_ = 0.009
559 parameters	Δρ_max_ = 0.30 e Å^−^^3^
3 restraints	Δρ_min_ = −0.27 e Å^−^^3^

### Special details

**Table:**

Geometry. All e.s.d.'s (except the e.s.d. in the dihedral angle between two l.s. planes) are estimated using the full covariance matrix. The cell e.s.d.'s are taken into account individually in the estimation of e.s.d.'s in distances, angles and torsion angles; correlations between e.s.d.'s in cell parameters are only used when they are defined by crystal symmetry. An approximate (isotropic) treatment of cell e.s.d.'s is used for estimating e.s.d.'s involving l.s. planes.
Refinement. Refinement of *F*^2^ against ALL reflections. The weighted *R*-factor *wR* and goodness of fit *S* are based on *F*^2^, conventional *R*-factors *R* are based on *F*, with *F* set to zero for negative *F*^2^. The threshold expression of *F*^2^ > σ(*F*^2^) is used only for calculating *R*-factors(gt) *etc*. and is not relevant to the choice of reflections for refinement. *R*-factors based on *F*^2^ are statistically about twice as large as those based on *F*, and *R*- factors based on ALL data will be even larger.

### Fractional atomic coordinates and isotropic or equivalent isotropic displacement parameters (Å^2^)

**Table:**

	*x*	*y*	*z*	*U*_iso_*/*U*_eq_	
C1	−0.0123 (4)	0.2933 (3)	0.5886 (2)	0.0407 (10)	
C2	0.0466 (4)	0.2210 (3)	0.6259 (2)	0.0461 (11)	
C3	0.0252 (5)	0.2188 (4)	0.6986 (2)	0.0566 (13)	
H3	0.0625	0.1719	0.7252	0.068*	
C4	−0.0491 (5)	0.2832 (4)	0.7326 (2)	0.0553 (12)	
C5	−0.1070 (4)	0.3535 (4)	0.6960 (2)	0.0560 (12)	
H5	−0.1581	0.3967	0.7193	0.067*	
C6	−0.0872 (4)	0.3583 (3)	0.6240 (2)	0.0480 (11)	
H6	−0.1246	0.4061	0.5984	0.058*	
C7	0.2848 (4)	0.4026 (3)	0.5328 (2)	0.0376 (10)	
C8	0.3452 (4)	0.4446 (3)	0.6042 (2)	0.0423 (10)	
C9	0.4730 (5)	0.4273 (4)	0.6240 (3)	0.0597 (13)	
H9	0.5167	0.4541	0.6712	0.072*	
C10	0.5370 (5)	0.3716 (4)	0.5761 (3)	0.0615 (14)	
H10	0.6226	0.3610	0.5917	0.074*	
C11	0.4779 (4)	0.3307 (3)	0.5051 (3)	0.0488 (12)	
C12	0.3515 (4)	0.3489 (3)	0.4846 (2)	0.0447 (11)	
H12	0.3099	0.3242	0.4368	0.054*	
C13	0.1285 (5)	0.1461 (4)	0.5926 (2)	0.0674 (14)	
H13A	0.2020	0.1888	0.5708	0.081*	
H13B	0.0699	0.0913	0.5558	0.081*	
H13C	0.1657	0.1105	0.6301	0.081*	
C14	0.2770 (5)	0.5061 (4)	0.6585 (2)	0.0593 (13)	
H14A	0.2060	0.4546	0.6765	0.071*	
H14B	0.2378	0.5577	0.6348	0.071*	
H14C	0.3440	0.5450	0.6986	0.071*	
C15	0.5475 (5)	0.2713 (4)	0.4519 (3)	0.0728 (15)	
H15A	0.5239	0.1939	0.4559	0.087*	
H15B	0.6451	0.2994	0.4626	0.087*	
H15C	0.5183	0.2823	0.4031	0.087*	
N1	0.1526 (3)	0.4174 (3)	0.50559 (18)	0.0403 (9)	
H1N	0.144 (4)	0.4799 (19)	0.521 (2)	0.048*	
O1	0.0410 (3)	0.2220 (2)	0.45847 (14)	0.0520 (8)	
O2	−0.0926 (3)	0.3631 (2)	0.46950 (14)	0.0524 (8)	
Cl1	−0.07089 (16)	0.27621 (13)	0.82370 (7)	0.0921 (5)	
S1	0.01622 (11)	0.31764 (9)	0.49840 (6)	0.0425 (3)	
C16	0.3635 (4)	0.3662 (3)	0.0733 (2)	0.0485 (11)	
C17	0.2916 (5)	0.4364 (4)	0.0417 (3)	0.0539 (12)	
C18	0.2890 (5)	0.4350 (4)	−0.0334 (3)	0.0619 (13)	
H18	0.2425	0.4800	−0.0565	0.074*	
C19	0.3527 (5)	0.3695 (4)	−0.0741 (3)	0.0624 (14)	
C20	0.4236 (5)	0.3014 (4)	−0.0430 (3)	0.0666 (14)	
H20	0.4674	0.2575	−0.0712	0.080*	
C21	0.4270 (5)	0.3009 (4)	0.0307 (3)	0.0589 (13)	
H21	0.4737	0.2552	0.0529	0.071*	
C22	0.1020 (5)	0.2799 (3)	0.1666 (2)	0.0422 (11)	
C23	0.0532 (5)	0.3311 (4)	0.2257 (2)	0.0552 (12)	
C24	−0.0736 (6)	0.3542 (4)	0.2122 (3)	0.0674 (14)	
H24	−0.1071	0.3916	0.2498	0.081*	
C25	−0.1512 (5)	0.3234 (4)	0.1452 (3)	0.0686 (15)	
H25	−0.2360	0.3405	0.1384	0.082*	
C26	−0.1064 (5)	0.2677 (4)	0.0878 (3)	0.0540 (12)	
C27	0.0213 (4)	0.2478 (3)	0.1001 (2)	0.0460 (11)	
H27	0.0546	0.2112	0.0621	0.055*	
C28	0.2215 (5)	0.5137 (4)	0.0837 (3)	0.0690 (14)	
H28A	0.2897	0.5729	0.1127	0.083*	
H28B	0.1639	0.4739	0.1152	0.083*	
H28C	0.1667	0.5433	0.0498	0.083*	
C29	0.1305 (6)	0.3550 (4)	0.3019 (2)	0.0848 (17)	
H29A	0.2103	0.4145	0.3037	0.102*	
H29B	0.1579	0.2904	0.3140	0.102*	
H29C	0.0721	0.3752	0.3364	0.102*	
C30	−0.1940 (5)	0.2269 (4)	0.0156 (3)	0.0870 (17)	
H30A	−0.2607	0.2696	0.0095	0.104*	
H30B	−0.2402	0.1508	0.0143	0.104*	
H30C	−0.1372	0.2341	−0.0233	0.104*	
N2	0.2349 (4)	0.2574 (3)	0.1770 (2)	0.0513 (10)	
H2N	0.234 (4)	0.194 (2)	0.154 (2)	0.062*	
O3	0.4835 (3)	0.3029 (3)	0.18490 (17)	0.0732 (10)	
O4	0.3632 (3)	0.4515 (2)	0.20869 (16)	0.0652 (9)	
Cl2	0.34232 (18)	0.37183 (14)	−0.16790 (8)	0.1049 (6)	
S2	0.37014 (13)	0.35082 (10)	0.16747 (7)	0.0552 (3)	
C31	0.3574 (4)	0.9718 (3)	0.2561 (2)	0.0466 (11)	
C32	0.4113 (4)	0.8992 (3)	0.2958 (3)	0.0499 (11)	
C33	0.3868 (5)	0.8999 (4)	0.3684 (3)	0.0578 (13)	
H33	0.4217	0.8533	0.3963	0.069*	
C34	0.3134 (5)	0.9665 (4)	0.4000 (2)	0.0556 (12)	
C35	0.2603 (4)	1.0378 (4)	0.3613 (3)	0.0577 (13)	
H35	0.2103	1.0833	0.3831	0.069*	
C36	0.2835 (4)	1.0395 (4)	0.2897 (3)	0.0523 (12)	
H36	0.2488	1.0872	0.2628	0.063*	
C37	0.6611 (4)	1.0536 (3)	0.1809 (2)	0.0452 (11)	
C38	0.7413 (5)	1.0907 (4)	0.2480 (3)	0.0535 (12)	
C39	0.8690 (5)	1.0663 (4)	0.2551 (3)	0.0646 (14)	
H39	0.9254	1.0886	0.2994	0.077*	
C40	0.9140 (5)	1.0096 (4)	0.1979 (3)	0.0672 (14)	
H40	1.0003	0.9952	0.2046	0.081*	
C41	0.8352 (5)	0.9738 (4)	0.1316 (3)	0.0556 (12)	
C42	0.7077 (5)	0.9977 (3)	0.1245 (2)	0.0510 (12)	
H42	0.6517	0.9751	0.0801	0.061*	
C43	0.4919 (5)	0.8212 (4)	0.2650 (3)	0.0727 (15)	
H43A	0.5670	0.8616	0.2432	0.087*	
H43B	0.4331	0.7658	0.2286	0.087*	
H43C	0.5267	0.7865	0.3037	0.087*	
C44	0.6944 (5)	1.1544 (4)	0.3089 (3)	0.0778 (16)	
H44A	0.6163	1.1084	0.3253	0.093*	
H44B	0.6695	1.2174	0.2920	0.093*	
H44C	0.7672	1.1786	0.3486	0.093*	
C45	0.8884 (5)	0.9164 (4)	0.0695 (3)	0.0869 (17)	
H45A	0.8625	0.8382	0.0704	0.104*	
H45B	0.9866	0.9410	0.0744	0.104*	
H45C	0.8502	0.9331	0.0240	0.104*	
N3	0.5293 (4)	1.0778 (3)	0.1664 (2)	0.0514 (10)	
H3N	0.520 (4)	1.143 (2)	0.179 (2)	0.062*	
O5	0.3991 (3)	0.8852 (2)	0.12759 (15)	0.0589 (8)	
O6	0.2813 (3)	1.0368 (2)	0.13558 (15)	0.0607 (9)	
Cl3	0.28700 (16)	0.96295 (12)	0.49092 (7)	0.0862 (5)	
S3	0.38552 (12)	0.98711 (10)	0.16454 (6)	0.0506 (3)	

### Atomic displacement parameters (Å^2^)

**Table:**

	*U*^11^	*U*^22^	*U*^33^	*U*^12^	*U*^13^	*U*^23^
C1	0.035 (3)	0.044 (3)	0.044 (3)	0.012 (2)	0.0019 (19)	0.005 (2)
C2	0.050 (3)	0.044 (3)	0.048 (3)	0.017 (2)	0.008 (2)	0.009 (2)
C3	0.071 (3)	0.054 (3)	0.052 (3)	0.022 (3)	0.009 (3)	0.020 (2)
C4	0.062 (3)	0.058 (3)	0.044 (3)	0.009 (3)	0.013 (2)	0.008 (2)
C5	0.054 (3)	0.066 (3)	0.053 (3)	0.020 (3)	0.017 (2)	0.006 (3)
C6	0.043 (3)	0.053 (3)	0.053 (3)	0.019 (2)	0.005 (2)	0.013 (2)
C7	0.033 (2)	0.038 (2)	0.044 (3)	0.011 (2)	0.000 (2)	0.013 (2)
C8	0.044 (3)	0.040 (3)	0.040 (3)	0.008 (2)	−0.003 (2)	0.007 (2)
C9	0.050 (3)	0.067 (3)	0.057 (3)	0.012 (3)	−0.014 (2)	0.008 (3)
C10	0.041 (3)	0.062 (3)	0.082 (4)	0.018 (3)	−0.009 (3)	0.011 (3)
C11	0.035 (3)	0.046 (3)	0.069 (3)	0.014 (2)	0.012 (2)	0.010 (2)
C12	0.043 (3)	0.045 (3)	0.045 (3)	0.009 (2)	0.002 (2)	0.009 (2)
C13	0.090 (4)	0.068 (3)	0.062 (3)	0.046 (3)	0.017 (3)	0.021 (3)
C14	0.069 (3)	0.065 (3)	0.039 (3)	0.014 (3)	−0.002 (2)	0.002 (2)
C15	0.046 (3)	0.072 (4)	0.104 (4)	0.022 (3)	0.018 (3)	0.004 (3)
N1	0.041 (2)	0.045 (2)	0.039 (2)	0.0186 (19)	0.0004 (16)	0.0073 (18)
O1	0.056 (2)	0.0540 (19)	0.0438 (18)	0.0178 (15)	−0.0007 (14)	−0.0040 (15)
O2	0.0422 (18)	0.067 (2)	0.0496 (18)	0.0214 (15)	−0.0093 (14)	0.0090 (15)
Cl1	0.1239 (13)	0.1087 (12)	0.0533 (8)	0.0349 (10)	0.0333 (8)	0.0205 (8)
S1	0.0377 (7)	0.0523 (7)	0.0378 (6)	0.0151 (6)	−0.0031 (5)	0.0058 (5)
C16	0.045 (3)	0.038 (3)	0.058 (3)	0.007 (2)	0.001 (2)	0.002 (2)
C17	0.059 (3)	0.043 (3)	0.062 (3)	0.013 (2)	0.013 (2)	0.009 (2)
C18	0.071 (4)	0.060 (3)	0.061 (3)	0.020 (3)	0.010 (3)	0.022 (3)
C19	0.070 (4)	0.057 (3)	0.055 (3)	0.001 (3)	0.018 (3)	0.008 (3)
C20	0.066 (4)	0.058 (3)	0.075 (4)	0.016 (3)	0.017 (3)	−0.005 (3)
C21	0.051 (3)	0.052 (3)	0.072 (4)	0.014 (2)	0.002 (3)	0.001 (3)
C22	0.057 (3)	0.033 (2)	0.034 (3)	0.006 (2)	0.002 (2)	0.0055 (19)
C23	0.073 (4)	0.050 (3)	0.041 (3)	0.010 (3)	0.011 (2)	0.009 (2)
C24	0.079 (4)	0.059 (3)	0.066 (4)	0.015 (3)	0.031 (3)	0.003 (3)
C25	0.052 (3)	0.066 (4)	0.093 (4)	0.018 (3)	0.014 (3)	0.020 (3)
C26	0.056 (3)	0.052 (3)	0.053 (3)	0.009 (3)	0.002 (3)	0.016 (2)
C27	0.057 (3)	0.042 (3)	0.042 (3)	0.015 (2)	0.008 (2)	0.008 (2)
C28	0.096 (4)	0.055 (3)	0.070 (3)	0.039 (3)	0.019 (3)	0.021 (3)
C29	0.124 (5)	0.085 (4)	0.037 (3)	0.010 (3)	0.013 (3)	0.004 (3)
C30	0.079 (4)	0.093 (4)	0.083 (4)	0.018 (3)	−0.022 (3)	0.020 (3)
N2	0.055 (3)	0.049 (2)	0.050 (2)	0.018 (2)	−0.0057 (19)	0.0043 (19)
O3	0.060 (2)	0.072 (2)	0.085 (3)	0.0257 (18)	−0.0225 (18)	0.0059 (18)
O4	0.079 (2)	0.051 (2)	0.056 (2)	0.0091 (17)	−0.0065 (17)	−0.0084 (16)
Cl2	0.1287 (14)	0.1233 (14)	0.0642 (10)	0.0251 (11)	0.0289 (9)	0.0179 (9)
S2	0.0559 (8)	0.0495 (8)	0.0555 (8)	0.0125 (6)	−0.0096 (6)	0.0016 (6)
C31	0.043 (3)	0.047 (3)	0.048 (3)	0.013 (2)	−0.003 (2)	0.002 (2)
C32	0.047 (3)	0.044 (3)	0.061 (3)	0.015 (2)	0.004 (2)	0.008 (2)
C33	0.066 (3)	0.053 (3)	0.059 (3)	0.019 (3)	0.006 (3)	0.015 (3)
C34	0.062 (3)	0.053 (3)	0.049 (3)	0.011 (3)	0.006 (2)	0.001 (2)
C35	0.059 (3)	0.057 (3)	0.058 (3)	0.021 (3)	0.007 (3)	−0.001 (3)
C36	0.044 (3)	0.055 (3)	0.059 (3)	0.021 (2)	−0.004 (2)	0.005 (2)
C37	0.043 (3)	0.040 (3)	0.052 (3)	0.008 (2)	0.001 (2)	0.013 (2)
C38	0.058 (3)	0.043 (3)	0.057 (3)	0.008 (2)	0.005 (3)	0.008 (2)
C39	0.058 (4)	0.060 (3)	0.066 (4)	0.001 (3)	−0.018 (3)	0.017 (3)
C40	0.051 (3)	0.058 (3)	0.097 (5)	0.014 (3)	0.009 (3)	0.025 (3)
C41	0.053 (3)	0.043 (3)	0.072 (4)	0.009 (2)	0.016 (3)	0.012 (3)
C42	0.052 (3)	0.051 (3)	0.050 (3)	0.010 (2)	0.006 (2)	0.009 (2)
C43	0.093 (4)	0.073 (4)	0.070 (4)	0.050 (3)	0.016 (3)	0.021 (3)
C44	0.090 (4)	0.073 (4)	0.058 (3)	0.008 (3)	−0.006 (3)	−0.006 (3)
C45	0.076 (4)	0.083 (4)	0.109 (5)	0.023 (3)	0.041 (3)	0.010 (3)
N3	0.057 (3)	0.043 (2)	0.056 (2)	0.017 (2)	0.0031 (19)	0.006 (2)
O5	0.063 (2)	0.053 (2)	0.057 (2)	0.0182 (16)	−0.0022 (15)	−0.0088 (16)
O6	0.054 (2)	0.074 (2)	0.056 (2)	0.0311 (17)	−0.0115 (15)	0.0013 (16)
Cl3	0.1112 (12)	0.0974 (11)	0.0588 (9)	0.0387 (9)	0.0215 (8)	0.0111 (8)
S3	0.0490 (8)	0.0533 (8)	0.0484 (7)	0.0170 (6)	−0.0040 (6)	0.0006 (6)

### Geometric parameters (Å, °)

**Table:**

C1—C6	1.389 (5)	C25—C26	1.377 (6)
C1—C2	1.405 (5)	C25—H25	0.9300
C1—S1	1.769 (4)	C26—C27	1.372 (6)
C2—C3	1.386 (5)	C26—C30	1.502 (6)
C2—C13	1.509 (5)	C27—H27	0.9300
C3—C4	1.367 (6)	C28—H28A	0.9600
C3—H3	0.9300	C28—H28B	0.9600
C4—C5	1.373 (6)	C28—H28C	0.9600
C4—Cl1	1.731 (4)	C29—H29A	0.9600
C5—C6	1.372 (5)	C29—H29B	0.9600
C5—H5	0.9300	C29—H29C	0.9600
C6—H6	0.9300	C30—H30A	0.9600
C7—C12	1.376 (5)	C30—H30B	0.9600
C7—C8	1.396 (5)	C30—H30C	0.9600
C7—N1	1.442 (5)	N2—S2	1.620 (4)
C8—C9	1.381 (5)	N2—H2N	0.853 (18)
C8—C14	1.511 (5)	O3—S2	1.439 (3)
C9—C10	1.369 (6)	O4—S2	1.424 (3)
C9—H9	0.9300	C31—C36	1.389 (5)
C10—C11	1.383 (6)	C31—C32	1.406 (5)
C10—H10	0.9300	C31—S3	1.770 (4)
C11—C12	1.374 (5)	C32—C33	1.388 (6)
C11—C15	1.493 (6)	C32—C43	1.510 (6)
C12—H12	0.9300	C33—C34	1.362 (6)
C13—H13A	0.9600	C33—H33	0.9300
C13—H13B	0.9600	C34—C35	1.379 (6)
C13—H13C	0.9600	C34—Cl3	1.733 (5)
C14—H14A	0.9600	C35—C36	1.369 (5)
C14—H14B	0.9600	C35—H35	0.9300
C14—H14C	0.9600	C36—H36	0.9300
C15—H15A	0.9600	C37—C42	1.374 (6)
C15—H15B	0.9600	C37—C38	1.388 (6)
C15—H15C	0.9600	C37—N3	1.441 (5)
N1—S1	1.618 (3)	C38—C39	1.391 (6)
N1—H1N	0.833 (18)	C38—C44	1.487 (6)
O1—S1	1.426 (3)	C39—C40	1.381 (6)
O2—S1	1.439 (3)	C39—H39	0.9300
C16—C21	1.379 (6)	C40—C41	1.370 (6)
C16—C17	1.409 (6)	C40—H40	0.9300
C16—S2	1.772 (4)	C41—C42	1.385 (6)
C17—C18	1.386 (6)	C41—C45	1.496 (6)
C17—C28	1.518 (6)	C42—H42	0.9300
C18—C19	1.363 (6)	C43—H43A	0.9600
C18—H18	0.9300	C43—H43B	0.9600
C19—C20	1.378 (6)	C43—H43C	0.9600
C19—Cl2	1.735 (5)	C44—H44A	0.9600
C20—C21	1.364 (6)	C44—H44B	0.9600
C20—H20	0.9300	C44—H44C	0.9600
C21—H21	0.9300	C45—H45A	0.9600
C22—C27	1.377 (5)	C45—H45B	0.9600
C22—C23	1.394 (6)	C45—H45C	0.9600
C22—N2	1.434 (5)	N3—S3	1.620 (4)
C23—C24	1.383 (6)	N3—H3N	0.853 (18)
C23—C29	1.513 (6)	O5—S3	1.426 (3)
C24—C25	1.370 (6)	O6—S3	1.435 (3)
C24—H24	0.9300		
			
C6—C1—C2	120.7 (4)	C25—C26—C30	122.0 (5)
C6—C1—S1	115.8 (3)	C26—C27—C22	122.6 (4)
C2—C1—S1	123.3 (3)	C26—C27—H27	118.7
C3—C2—C1	116.4 (4)	C22—C27—H27	118.7
C3—C2—C13	118.5 (4)	C17—C28—H28A	109.5
C1—C2—C13	125.0 (4)	C17—C28—H28B	109.5
C4—C3—C2	122.1 (4)	H28A—C28—H28B	109.5
C4—C3—H3	118.9	C17—C28—H28C	109.5
C2—C3—H3	118.9	H28A—C28—H28C	109.5
C3—C4—C5	121.5 (4)	H28B—C28—H28C	109.5
C3—C4—Cl1	119.4 (4)	C23—C29—H29A	109.5
C5—C4—Cl1	119.1 (4)	C23—C29—H29B	109.5
C6—C5—C4	118.0 (4)	H29A—C29—H29B	109.5
C6—C5—H5	121.0	C23—C29—H29C	109.5
C4—C5—H5	121.0	H29A—C29—H29C	109.5
C5—C6—C1	121.4 (4)	H29B—C29—H29C	109.5
C5—C6—H6	119.3	C26—C30—H30A	109.5
C1—C6—H6	119.3	C26—C30—H30B	109.5
C12—C7—C8	121.2 (4)	H30A—C30—H30B	109.5
C12—C7—N1	117.3 (4)	C26—C30—H30C	109.5
C8—C7—N1	121.5 (4)	H30A—C30—H30C	109.5
C9—C8—C7	116.6 (4)	H30B—C30—H30C	109.5
C9—C8—C14	120.5 (4)	C22—N2—S2	119.8 (3)
C7—C8—C14	123.0 (4)	C22—N2—H2N	110 (3)
C10—C9—C8	121.7 (4)	S2—N2—H2N	112 (3)
C10—C9—H9	119.2	O4—S2—O3	119.7 (2)
C8—C9—H9	119.2	O4—S2—N2	107.81 (19)
C9—C10—C11	121.9 (4)	O3—S2—N2	104.9 (2)
C9—C10—H10	119.1	O4—S2—C16	109.3 (2)
C11—C10—H10	119.1	O3—S2—C16	107.2 (2)
C12—C11—C10	116.9 (4)	N2—S2—C16	107.28 (19)
C12—C11—C15	120.8 (4)	C36—C31—C32	120.1 (4)
C10—C11—C15	122.4 (4)	C36—C31—S3	116.9 (3)
C11—C12—C7	121.8 (4)	C32—C31—S3	123.0 (3)
C11—C12—H12	119.1	C33—C32—C31	116.7 (4)
C7—C12—H12	119.1	C33—C32—C43	118.5 (4)
C2—C13—H13A	109.5	C31—C32—C43	124.8 (4)
C2—C13—H13B	109.5	C34—C33—C32	122.1 (4)
H13A—C13—H13B	109.5	C34—C33—H33	118.9
C2—C13—H13C	109.5	C32—C33—H33	118.9
H13A—C13—H13C	109.5	C33—C34—C35	121.3 (4)
H13B—C13—H13C	109.5	C33—C34—Cl3	119.6 (4)
C8—C14—H14A	109.5	C35—C34—Cl3	119.1 (4)
C8—C14—H14B	109.5	C36—C35—C34	117.9 (4)
H14A—C14—H14B	109.5	C36—C35—H35	121.1
C8—C14—H14C	109.5	C34—C35—H35	121.1
H14A—C14—H14C	109.5	C35—C36—C31	121.9 (4)
H14B—C14—H14C	109.5	C35—C36—H36	119.1
C11—C15—H15A	109.5	C31—C36—H36	119.1
C11—C15—H15B	109.5	C42—C37—C38	121.3 (4)
H15A—C15—H15B	109.5	C42—C37—N3	117.4 (4)
C11—C15—H15C	109.5	C38—C37—N3	121.3 (4)
H15A—C15—H15C	109.5	C37—C38—C39	116.4 (5)
H15B—C15—H15C	109.5	C37—C38—C44	122.0 (5)
C7—N1—S1	120.8 (3)	C39—C38—C44	121.5 (5)
C7—N1—H1N	111 (3)	C40—C39—C38	121.6 (5)
S1—N1—H1N	116 (3)	C40—C39—H39	119.2
O1—S1—O2	118.96 (17)	C38—C39—H39	119.2
O1—S1—N1	108.79 (17)	C41—C40—C39	121.9 (5)
O2—S1—N1	104.42 (17)	C41—C40—H40	119.0
O1—S1—C1	109.92 (18)	C39—C40—H40	119.0
O2—S1—C1	107.45 (18)	C40—C41—C42	116.6 (5)
N1—S1—C1	106.54 (18)	C40—C41—C45	120.9 (5)
C21—C16—C17	120.7 (4)	C42—C41—C45	122.5 (5)
C21—C16—S2	116.5 (4)	C37—C42—C41	122.2 (4)
C17—C16—S2	122.8 (4)	C37—C42—H42	118.9
C18—C17—C16	116.2 (4)	C41—C42—H42	118.9
C18—C17—C28	118.8 (4)	C32—C43—H43A	109.5
C16—C17—C28	125.0 (4)	C32—C43—H43B	109.5
C19—C18—C17	121.8 (5)	H43A—C43—H43B	109.5
C19—C18—H18	119.1	C32—C43—H43C	109.5
C17—C18—H18	119.1	H43A—C43—H43C	109.5
C18—C19—C20	121.9 (5)	H43B—C43—H43C	109.5
C18—C19—Cl2	118.7 (4)	C38—C44—H44A	109.5
C20—C19—Cl2	119.4 (4)	C38—C44—H44B	109.5
C21—C20—C19	117.3 (5)	H44A—C44—H44B	109.5
C21—C20—H20	121.3	C38—C44—H44C	109.5
C19—C20—H20	121.3	H44A—C44—H44C	109.5
C20—C21—C16	122.0 (5)	H44B—C44—H44C	109.5
C20—C21—H21	119.0	C41—C45—H45A	109.5
C16—C21—H21	119.0	C41—C45—H45B	109.5
C27—C22—C23	120.2 (4)	H45A—C45—H45B	109.5
C27—C22—N2	120.7 (4)	C41—C45—H45C	109.5
C23—C22—N2	119.0 (4)	H45A—C45—H45C	109.5
C24—C23—C22	116.8 (4)	H45B—C45—H45C	109.5
C24—C23—C29	121.0 (5)	C37—N3—S3	122.9 (3)
C22—C23—C29	122.1 (5)	C37—N3—H3N	120 (3)
C25—C24—C23	121.9 (5)	S3—N3—H3N	111 (3)
C25—C24—H24	119.0	O5—S3—O6	119.22 (18)
C23—C24—H24	119.0	O5—S3—N3	107.91 (18)
C24—C25—C26	121.4 (5)	O6—S3—N3	105.51 (19)
C24—C25—H25	119.3	O5—S3—C31	109.50 (19)
C26—C25—H25	119.3	O6—S3—C31	106.40 (19)
C27—C26—C25	117.0 (5)	N3—S3—C31	107.79 (19)
C27—C26—C30	121.0 (5)		
			
C6—C1—C2—C3	0.2 (6)	C23—C24—C25—C26	0.0 (7)
S1—C1—C2—C3	174.0 (3)	C24—C25—C26—C27	−2.1 (7)
C6—C1—C2—C13	179.4 (4)	C24—C25—C26—C30	175.9 (4)
S1—C1—C2—C13	−6.9 (6)	C25—C26—C27—C22	0.9 (6)
C1—C2—C3—C4	−0.1 (7)	C30—C26—C27—C22	−177.1 (4)
C13—C2—C3—C4	−179.3 (4)	C23—C22—C27—C26	2.5 (6)
C2—C3—C4—C5	0.3 (7)	N2—C22—C27—C26	−180.0 (4)
C2—C3—C4—Cl1	−179.7 (4)	C27—C22—N2—S2	95.4 (4)
C3—C4—C5—C6	−0.6 (7)	C23—C22—N2—S2	−87.0 (4)
Cl1—C4—C5—C6	179.4 (3)	C22—N2—S2—O4	48.9 (4)
C4—C5—C6—C1	0.7 (7)	C22—N2—S2—O3	177.5 (3)
C2—C1—C6—C5	−0.6 (6)	C22—N2—S2—C16	−68.7 (4)
S1—C1—C6—C5	−174.8 (3)	C21—C16—S2—O4	152.3 (3)
C12—C7—C8—C9	−1.3 (6)	C17—C16—S2—O4	−31.1 (4)
N1—C7—C8—C9	−178.9 (4)	C21—C16—S2—O3	21.1 (4)
C12—C7—C8—C14	178.7 (4)	C17—C16—S2—O3	−162.2 (3)
N1—C7—C8—C14	1.1 (6)	C21—C16—S2—N2	−91.1 (4)
C7—C8—C9—C10	−0.1 (6)	C17—C16—S2—N2	85.6 (4)
C14—C8—C9—C10	179.9 (4)	C36—C31—C32—C33	−0.2 (6)
C8—C9—C10—C11	0.7 (7)	S3—C31—C32—C33	176.8 (3)
C9—C10—C11—C12	0.2 (7)	C36—C31—C32—C43	179.4 (4)
C9—C10—C11—C15	179.1 (4)	S3—C31—C32—C43	−3.7 (6)
C10—C11—C12—C7	−1.7 (6)	C31—C32—C33—C34	0.5 (7)
C15—C11—C12—C7	179.4 (4)	C43—C32—C33—C34	−179.0 (4)
C8—C7—C12—C11	2.3 (6)	C32—C33—C34—C35	−0.5 (7)
N1—C7—C12—C11	180.0 (4)	C32—C33—C34—Cl3	180.0 (3)
C12—C7—N1—S1	79.7 (4)	C33—C34—C35—C36	0.1 (7)
C8—C7—N1—S1	−102.6 (4)	Cl3—C34—C35—C36	179.6 (3)
C7—N1—S1—O1	−51.7 (3)	C34—C35—C36—C31	0.3 (7)
C7—N1—S1—O2	−179.7 (3)	C32—C31—C36—C35	−0.3 (7)
C7—N1—S1—C1	66.8 (3)	S3—C31—C36—C35	−177.4 (3)
C6—C1—S1—O1	−156.3 (3)	C42—C37—C38—C39	−1.3 (6)
C2—C1—S1—O1	29.7 (4)	N3—C37—C38—C39	−177.5 (4)
C6—C1—S1—O2	−25.4 (4)	C42—C37—C38—C44	178.0 (4)
C2—C1—S1—O2	160.5 (3)	N3—C37—C38—C44	1.8 (6)
C6—C1—S1—N1	86.0 (3)	C37—C38—C39—C40	1.0 (7)
C2—C1—S1—N1	−88.0 (4)	C44—C38—C39—C40	−178.3 (4)
C21—C16—C17—C18	0.1 (6)	C38—C39—C40—C41	−0.5 (7)
S2—C16—C17—C18	−176.4 (3)	C39—C40—C41—C42	0.2 (7)
C21—C16—C17—C28	−178.2 (4)	C39—C40—C41—C45	177.6 (4)
S2—C16—C17—C28	5.2 (6)	C38—C37—C42—C41	1.1 (6)
C16—C17—C18—C19	−0.1 (7)	N3—C37—C42—C41	177.4 (4)
C28—C17—C18—C19	178.4 (4)	C40—C41—C42—C37	−0.4 (6)
C17—C18—C19—C20	−0.3 (8)	C45—C41—C42—C37	−177.9 (4)
C17—C18—C19—Cl2	179.2 (3)	C42—C37—N3—S3	78.6 (5)
C18—C19—C20—C21	0.6 (7)	C38—C37—N3—S3	−105.0 (4)
Cl2—C19—C20—C21	−178.9 (3)	C37—N3—S3—O5	−41.3 (4)
C19—C20—C21—C16	−0.5 (7)	C37—N3—S3—O6	−169.8 (3)
C17—C16—C21—C20	0.2 (7)	C37—N3—S3—C31	76.9 (4)
S2—C16—C21—C20	176.9 (4)	C36—C31—S3—O5	−152.0 (3)
C27—C22—C23—C24	−4.4 (6)	C32—C31—S3—O5	31.0 (4)
N2—C22—C23—C24	178.0 (4)	C36—C31—S3—O6	−21.9 (4)
C27—C22—C23—C29	172.3 (4)	C32—C31—S3—O6	161.1 (3)
N2—C22—C23—C29	−5.3 (6)	C36—C31—S3—N3	90.9 (4)
C22—C23—C24—C25	3.2 (7)	C32—C31—S3—N3	−86.1 (4)
C29—C23—C24—C25	−173.5 (4)		

### Hydrogen-bond geometry (Å, °)

**Table:**

*D*—H···*A*	*D*—H	H···*A*	*D*···*A*	*D*—H···*A*
N1—H1N···O2^i^	0.83 (2)	2.15 (2)	2.958 (4)	164 (4)
N2—H2N···O6^ii^	0.85 (2)	2.15 (2)	2.951 (5)	157 (4)
N3—H3N···O3^iii^	0.85 (2)	2.13 (2)	2.962 (5)	166 (4)

Symmetry codes: (i) −*x*, −*y*+1, −*z*+1; (ii) *x*, *y*−1, *z*; (iii) *x*, *y*+1, *z*.
